# Detection of Epstein Barr Virus by Chromogenic In Situ Hybridization in cases of extra-hepatic biliary atresia

**DOI:** 10.1186/1746-1596-3-19

**Published:** 2008-04-28

**Authors:** Fatemeh Mahjoub, Reza Shahsiah, Farid Azmoudeh Ardalan, Guiti Iravanloo, Mehri Najafi Sani, Abdolmajid Zarei, Maryam Monajemzadeh, Fatemeh Farahmand, Setareh Mamishi

**Affiliations:** 1 Pathology Department, Markaze Tebbi Koodakan (Children Hospital related to Tehran University of Medical Sciences), End of Keshavarz Boulevard, Tehran, Iran; 2 Department of Pathology and Laboratory Medicine, Imam Khomeini Hospital, Tehran University of Medical Sciences, Tehran, Iran; 3 Department of Gastro – Enterology, Markaze Tebbi Koodakan (Children Hospital related to Tehran University of Medical Sciences), End of Keshavarz Boulevard, Tehran, Iran; 4 Infectious Disease Research Center, Markaze Tebbi Koodakan (Children Hospital related to Tehran University of Medical Sciences), End of Keshavarz Boulevard, Tehran, Iran

## Abstract

**Introduction:**

Extra-hepatic biliary atresia (EHBA) is an important cause of neonatal cholestasis. Several infectious agents have been proposed as etiologic factors such as Rotavirus and Reovirus. There is limited data on the role of Epstein Barr virus (EBV) infection in EHBA, so we decided to study the presence of EBV virus in a series of 16 proven EHBA cases by Chromogenic in situ hybridization (CISH) technique.

**Methods:**

In the current study a total of 16 liver wedge biopsies of proven cases of EHBA were selected in a period of 4 years. CISH staining for EBV-encoded RNA (EBER) transcript was performed.

**Results:**

The review of H&E-stained slides of liver biopsies revealed fibrosis and marked ductular proliferation. In CISH-stained slides, EBV trace was observed in hepatocytes in two cases and in biliary epithelium in one case of EHBA.

**Discussion:**

Considering the association of hepatitis with the Epstein-Barr virus in later life, it is likely that EBV hepatitis and its complications occur in the neonatal/perinatal period. Since EHBA is a relatively rare disease, a similar study on wedge biopsies of this number of proven cases of EHBA has not been performed to date. Current observation proposes the need for a study of larger series and employing other methods for confirming the etiologic role of EBV in EHBA cases.

## Introduction

Extrahepatic biliary atresia can be defined as a progressive necroinflammatory process involving a segment or the entire extrahepatic biliary tree leading to loss of patency of the lumen and obstruction to bile flow [1]. EHBA occurs in 8 in 15000 live births resulting in 250 to 400 new cases per year in the USA [1]. Although we don't have a documented incidence rate of the disease in Iran, we encounter these cases frequently, especially in summer and winter. The pathogenesis of EHBA remains a mystery, though most of the causal theories and research to date can be grouped into four main categories: infectious or toxin exposure, abnormal morphogenesis, genetic predisposition and immune dysregulation [1]. Reovirus and Rotavirus particles have been found in liver and bile duct remnants of patients with EHBA [2,3]. Evidence for human papilloma virus (HPV) types 6 and 18 has also been reported [4]. There is no evidence for a causative role by hepatitis B or C in the disease [5]. The available data about EBV is rather limited, but there is a serologically documented case on record [6]. However, EBV-associated hepatitis is well recognized [7].

EBV is a member of the herpesvirus family. As with other herpesviruses, EBV is an enveloped virus that contains a DNA core surrounded by an icosahedral nucleocapsid and infection is endemic around the world [8]. It is now known that EBV infects 90% of the world's adult population. Upon infection, the individual remains a lifelong carrier of the virus [8]. Available detection methods for EBV are: PCR, in situ hybridization (ISH) and immunohistochemistry (IHC). ISH is the standard procedure for detecting EBV-encoded RNAs (EBERs) [9]. According to some authors, PCR and chromogenic in situ hybridization (CISH) are equally sensitive in detecting EBV in routinely processed liver biopsies, while IHC is an insensitive method [9]. Although larger biopsies are generally needed for CISH, in contrast to PCR, it allows identification and distinction of infected cell types [9]. This is generally considered an advantage, since the correct diagnosis of EBV hepatitis requires detection of the EBERs in parenchymal cells, not periportal lymphocytes [10]. The ready implementation of ISH in pathology laboratories makes it a useful ancillary tool in confirming the diagnosis of EBV infection in equivocal cases. This technique is based on detection of EBV-encoded RNAs (EBERs). EBER 1 and 2 are nonpolyadenylated, uncapped, noncoding RNAs of 167 and 172 nucleotides respectively, and are expressed abundantly in nearly all EBV-infected cells [11]. Peptide nucleic acid (PNA) probes are usually employed for hybridization. PNA molecules are DNA mimics, where the negatively charged sugar-phosphate backbone of DNA is replaced by a neutral polyamide backbone formed by repetitive units of N-(2-aminoethyl) glycine [12]. Individual nucleotide bases are attached to each of the units to provide a molecular design that enables PNA to hybridize to complementary nucleic acid targets according to the Watson and Crick base-pairing rules. The synthetic backbone provides PNA probes with unique hybridization characteristics, such as more rapid and stronger binding to complementary targets [13].

EBV infection is seen in Iran rather commonly and we suggest a role for it at least in a fraction of EHBA cases. There is limited data in EHBA on the role of EBV infection, so we decided to study the presence of EBV virus in a series of 16 proven EHBA cases by CISH technique.

## Materials and methods

A total of 17 liver wedge biopsies were selected in a period of 4 years from 2004 to 2008 in Markaze Tebbi Koodakan. All patients had confirmation of extrahepatic biliary artesia by intraoperative cholangiogaphy and underwent Kasai operation subsequently. Liver wedge biopsies were taken just before the Kasai operation. Patients records were studied for clinical and biochemical data. H&E-stained slides and formalin-fixed paraffin-embedded blocks were available for study. CISH staining for EBV encoded RNA (EBER) transcripts were performed on standard 5-μm deparaffinized tissue sections mounted on silanized slides. Slides were digested with proteinase K for 30 minutes, incubated with Fluorescein isothiocyanate (FITC)-conjugated EBER PNA-probes (DAKO code-Nr.Y 5200), followed by incubation with anti-FITC alkaline phosphatase-conjugated antibody and 4-nitroblue tetrazolium chloride/5-bromo-4-chloro-3-indolyl phosphate substrate combined with levamisole (DAKO code-Nr. K 5201). Manufacturer's instructions were followed carefully, and contamination with RNAses was avoided strictly. Glassware was made RNAse-free by an overnight incubation at 200°C, and Diethyl Pyrocarbonate (DPEC)-treated water was used for preparation of reagents. Cells exhibiting nuclear staining were considered positive. Positive and negative controls were included in each run as recommended by the manufacturer.

## Results

**Figure 1:**
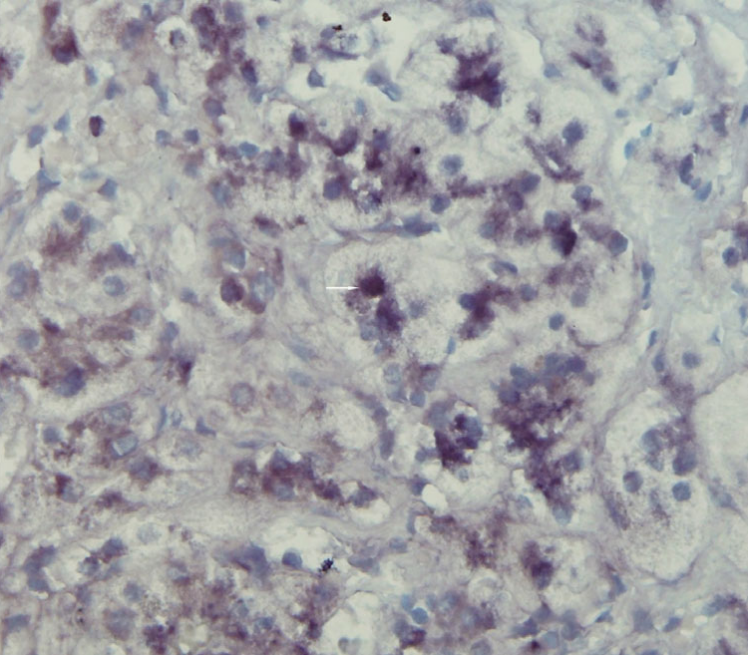
Detection of EBV by CISH. EBER-positive hepatocytes (dark nuclei) were detected in this case (arrow), (original magnification × 400).

**Figure 2:**
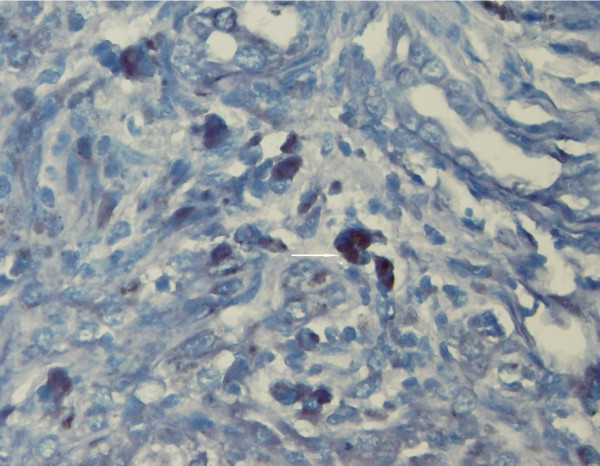
Detection of EBV by CISH. EBER-positive cholangiocytes (dark nuclei) were detected in this case (arrow), (original magnification × 400).

Of 17 selected patients, one has limited tissue and was excluded from study. Of 16 remaining patients, ten (59%) were male, and the rest (41%) were female. Age of patients at time of admission ranged from 36 to 152 days with mean and standard deviation of 72 and 39 days respectively. Seasonal distribution of the patients had two peaks, one in summer and another in winter. Total bilirubin ranged from 4.4 to 12.7 mg/dL with mean and standard deviation of 9.53 and 2.75 mg/dL, respectively. Alkaline Phosphatase ranged from 919 to 2703 IU/dL with mean and standard deviation of 1585 and 469 IU/dL, respectively. Aspartate aminotransferase (AST) ranged from 92 to 507 IU/dl with mean and standard deviation of 296 and 144 respectively. Alanine Aminotransferase (ALT) ranged from 40 to 475 IU/dl with mean and standard deviation of 158 and 108, respectively (Table 1). The review of H&E-stained slides of liver biopsies revealed fibrosis and marked ductular proliferation in all but one case which showed minimal ductular proliferation. Reviewing the CISH slides showed EBER-positive hepatocytes in two patients (Figure 1), and in biliary epithelium in one case (Figure 2). Another slide revealed periportal lymphocytes positive for EBER. Confirmation by serology studies was not performed.

**Table 1:** Gender and Age of admission and other paraclinical data.

**ID**	**Gender**	**Age(days)**	**ALP**	**AST**	**ALT**
1	F	70	1705	184	83
2	M	36	972	426	475
3	F	113	1514	374	116
4	M	52	1421	N/A	N/A
5	M	152	1451	486	209
6	F	114	N/A	420	204
7	M	37	2703	507	157
8	F	85	919	92	112
9	F	28	1697	227	70
10	M	74	N/A	N/A	N/A
11	M	72	N/A	207	197
12	F	72	N/A	N/A	N/A
13	M	90	1246	276	128
14	M	39	1657	175	129
15	M	28	1898	92	40
16	F	84	1831	385	134

ALP: Alkaline phosphatase, AST: Aspartate aminotransferase, ALT: Alanine aminotransferase.

## Discussion

EHBA is an important cause of neonatal cholestasis. Several infectious agents have been proposed such as Rotavirus and Reovirus [2,3]. The available data about EBV is rather limited, but there is a serologically documented case on record [6]. Although EBV hepatitis has been reported in some articles [7], there is no published article about EBV in liver biopsies in EHBA. The correct diagnosis of EBV hepatitis requires detection of the EBERs in parenchymal cells, not periportal lymphocytes, since some B cells in the circulation are infected with EBV [10]. However, EBER-positive cells can be sparse and easily overlooked [10], therefore only wedge biopsies were included in the study. Considering the association of hepatitis with the Epstein-Barr virus in later life, it is likely that EBV hepatitis and its complications occur in the neonatal/perinatal period. Also, cholestatic jaundice has been well-documented in patients with EBV hepatitis [14,15]. Even though the underlying mechanisms for EBV-induced cholestasis are not known, one possibility is that EBV may serve as the trigger for an autoimmune reaction in these cases. This is a phenomenon that has been described in genetically predisposed individuals [16].

A high number of EBV-positive cases were not expected from the very beginning of the study; meanwhile only 17 wedge biopsies were available in our referral center in the specified period of time. A case-series was designed, instead of a case-control study, simply because much more cases are needed to prove or rule out the causative role of EBV in EHBA using case-control method. On the other hand, EBV trace was found in three cases, two in hepatocytes and one in biliary epithelium, a feature that has not been described previously and still is worthy of recording. This proposes the need for a study of a larger series and employing other methods for confirming or ruling out the etiologic role of EBV in EHBA cases.

Drawbacks of the current study other than a limited number of cases include unavailability of serologic data and PCR for EBV infection. However according to N. Suh et al. PCR and ISH are equally sensitive in detecting EBV in routinely processed liver biopsies [10]. Since EHBA is relatively rare disease, a similar study on wedge biopsies of this number of proven cases of EHBA has not been performed to date.

## Conclusion

CISH staining was performed on liver wedge biopsies of 16 proven cases of EHBA, which represents the largest series for a biopsy-based study on EBV infection in this disorder. Hepatocytes were found to be positive in two cases and one case revealed positivity in biliary epithelium. This observation proposes the need for a study of a larger series and employing other methods for confirming the etiologic role of EBV in EHBA cases. Whether the EBV infection is responsible for the development of some cases of EHBA or not, remains to be proved in future studies performed on larger series.

## References

[B1] Russo P, Barness EG (2007). Liver including tumors, gallbladder, and biliary tree. Potter's Pathology of Fetus and Infant.

[B2] Morecki R, Glaser JH, Cho S, Balistreri WF, Horwitz MS (1982). Biliary atresia and reovirus type 3 infection. New England Journal of Medicine.

[B3] Rosenthal P (1995). The association of reovirus 3 and biliary atresia: finally resolved?. American Journal of Gastroenterology.

[B4] Drut R, Drut RM, Gomez MA, Cueto Rua E, Lojo MM (1998). Presence of human papillomavirus in extrahepatic biliary atresia. Journal of Pediatric Gastroenterology.

[B5] A-Kader HH, Nowicki MJ, Kuramoto KI, Baroudy B, Zeldis JB, Balistreri WF (1994). Evaluation of the role of hepatitis C virus in biliary atresia. Pediatric Infectous Disease.

[B6] Weaver LT, Nelson R, Bell TM (1984). The association of extrahepatic bile duct atresia and neonatal Epstein-Barr virus infection. Acta Paediatrica Scandinavica.

[B7] Crum NF (2006). Epstein Barr virus hepatitis: case series and review. South Medical Journal.

[B8] Cohen JI (2000). Epstein-Barr virus infection. New England Journal of Medicine.

[B9] Hassan R, White LR, Stefanoff CG, de Oliveira DE, Felisbino FE, Klumb CE, Bacchi CE, Seuánez HN, Zalcberg IR (2006). Epstein-Barr virus (EBV) detection and typing by PCR: a contribution to diagnostic screening of EBV-positive Burkitt's lymphoma. Diagnostic Pathology.

[B10] Suh N, Liapis H, Misdraji J, Brunt EM, Wang HL (2007). Epstein-Barr Virus Hepatitis: Diagnostic Value of In Situ Hybridization, Polymerase Chain Reaction, and Immunohistochemistry on Liver Biopsy From Immunocompetent Patients. The American Journal of Surgical Pathology.

[B11] Glickman JN, Howe JG, Steitz JA (1988). Structural analyses of EBER1 and EBER2. ribonucleoprotein particles present in Epstein-Barr virus-infected cells. Journal of Virology.

[B12] Nielsen E, Egholm M, Buchard O (1994). Peptide Nucleic Acid (PNA), a DNA mimic with a peptide backbone. Bioconjugate Chemistry.

[B13] Egholm M, Buchardt O, Christensen L, Behrens C, Freier SM, Driver DA, Berg RH, Kim SK, Norden B, Nielsen PE (1993). PNA hybridizes to complementary oligonucleotides obeying the Watson-Crick hydrogen bonding rules. Nature.

[B14] Markin RS (1994). Manifestations of Epstein-Barr virus-associated disorders in liver. Liver.

[B15] Edoute Y, Baruch Y, Lachter J, Furman E, Bassan L, Assy N (1998). Severe cholestatic jaundice induced by Epstein-Barr virus infection in the elderly. Journal of Gastroenterolgy and Hepatology.

[B16] Kojima K, Nagayama R, Hirama S, Maeda1 T, Takikawa1 H, Miyake1 K, Yamanaka1 M, Shiga J (1999). Epstein-Barr virus infection resembling autoimmune hepatitis with lactate dehydrogenase and alkaline phosphatase anomaly. Journal of Gastroenterolgy.

